# Spatial dynamics of cancer-associated fibroblasts links fibroblastic differentiation to immune exclusion and tumor progression

**DOI:** 10.3389/fimmu.2026.1886779

**Published:** 2026-08-28

**Authors:** Lingyi Cai, Tai-Hsien Ou Yang, Dimitris Anastassiou

**Affiliations:** 1 Department of Systems Biology, Columbia University, NY, United States; 2 Department of Electrical Engineering, Columbia University, NY, United States; 3 Herbert Irving Comprehensive Cancer Center, Columbia University, NY, United States

**Keywords:** cancer-associated fibroblasts, Immune exclusion, spatial modeling, Spatial transcriptomics, Tumor micro-environment

## Abstract

**Introduction:**

Although the role of cancer-associated fibroblasts (CAFs) in cancer progression is increasingly recognized, their spatial dynamics and interactions with immune cells remain poorly understood.

**Methods:**

Here, we present a computational framework that integrates single-cell resolution spatial transcriptomics and standard spatial transcriptomics across multiple tumor types to investigate CAF heterogeneity and its roles *in situ*.

**Results:**

Our analysis presents a continuous transition from fibroblast progenitors to COL11A1-expressing CAFs, which we term aggressive CAFs (aCAFs), within a spatial context. We show that aCAFs, whose expression has been associated with poor prognosis, tend to localize at tumor boundaries, where proximity to tumor cells predicts increased expression of aCAF-associated genes. Spatial modeling shows that regions enriched for COL11A1-expressing CAFs were depleted of non-exhausted immune cells, including naive T cells, activated cytotoxic T cells, and activated B cells, suggesting a role in immune exclusion. Spatial correlation analysis further reveals that aCAFs co-localize with lipid-associated macrophages, a pattern linked to extracellular matrix remodeling and altered lipid metabolism.

**Conclusion:**

Our study provides insights into the interactions of aCAF, tumor cells, and immune cells in the tumor microenvironment. We also provide an open-source implementation of SpatialAttractor, a toolkit for exploring gene co-expression in the spatial context.

## 1 Introduction

Cancer-associated fibroblasts (CAFs) play important roles in the tumor microenvironment (TME), remodeling the extracellular matrix (ECM), and modulating immune responses in ways that influence progression and therapeutic outcomes (1–4). CAFs are often organized into several populations, including myofibroblastic CAFs (myCAFs), inflammatory CAFs (iCAFs), and antigen-presenting CAFs (apCAFs) (1, 2, 5). Among CAF subpopulations, those myCAFs expressing COL11A1 have been found to be associated with tumor aggressiveness, metastasis, chemoresistance, and poor prognosis across multiple cancers (6–9). We refer to these COL11A1-expressing CAFs as “aggressive CAFs” (aCAFs) and quantify them using a gene-expression signature defined from the top ten genes in Kim et al. (6), including genes COL11A1, THBS2, COL10A1, COL5A2, INHBA, LRRC15, COL5A1, VCAN, FAP, and COL1A1, to which we refer as the “aCAF metagene.” The signature had been found to be stage-associated in a cancer type specific manner. For example, it never appears in ovarian cancer before reaching stage III, in colon cancer before reaching stage II and in breast carcinoma *in situ* (stage 0) before reaching invasive stage I. This suggests that the formation of aCAFs is triggered by the interaction of cancer cells with a particular cell population.

Previous analysis of single-cell RNA-seq datasets across diverse cancer types and normal samples suggested that aCAFs derive from a continuous transition from universal fibroblast progenitors and the expression of COL11A1 corresponds to the endpoint of the transition (7). The corresponding gene signature has been identified as characterizing a fibro-adipogenic progenitor (FAP) population with both adipogenic and fibroblastic differentiation potential (10), found mostly present in the stromal vascular fraction (SVF) of adipose tissue as adipose stromal cells (ASCs). In this study, we refer to this population and transition as the ASC/FAP population and the ASC/FAP-to-aCAF transition, respectively. This progenitor population is characterized by another well-defined signature including genes such as APOD, DCN, LUM, CFD, CXCL14, PTGDS, MGP, SERPINF1, and DPT (7, 10). These results suggest that the aCAF program reflects tumor cell interaction with ASC/FAPs, consistent with the tumor stage association of aCAFs. These analyses, however, lacked spatial context and did not resolve how this program relates to local immune composition and function.

Recent spatial transcriptomic studies across multiple tumor types have reported that SPP1-expressing tumor-associated macrophages (SPP1+ TAMs) co-localize with ECM-remodeling CAF subsets characterized by the aCAF signature (e.g., POSTN+, LRRC15+, or COL11A1+) and are associated with reduced T cell infiltration and features of immune suppression in different cancer types (11–18). As an example, Yan et al. (11) reported that tumor cells and SPP1+ macrophages interact with COL11A1+ CAFs to stimulate the deposition and entanglement of collagen fibers at tumor boundaries, obstructing T cell infiltration and leading to poor prognosis. These observations point to a pattern of macrophage-aCAF co-localization across multiple cancer types (3, 19). Beyond the immune-CAF co-localization, stromal cells can also form physical barriers through excessive ECM deposition and remodeling that restrict immune-cell entry (11, 20, 21). At tumor margins, COL11A1+ CAFs and SPP1+ macrophages have been reported to interact with tumor cells to promote collagen deposition and entanglement, forming a dense boundary associated with immune exclusion and immunotherapy resistance (11). Similar tumor-stroma barrier architectures have been linked to ECM-producing CAF programs and reduced immune infiltration in various cancer types (22–29). However, several issues have not been fully addressed, such as the spatial patterns of the transition of ASC/FAPs into fully differentiated aCAFs across cancers, the functional characterization of the immune cell populations that are co-localized with aCAFs, how tumor proximity relates to aCAF differentiation and accumulation, and which immune cell populations are blocked at the tumor-stroma barrier.

Here, we integrate single-cell-resolution spatial transcriptomics with spot-resolution transcriptomics datasets across multiple tumor types, validating the ASC/FAP-to-aCAF transition *in situ* and characterizing its interactions with the immune cell populations. Our work (a) reconstructs a spatial ASC/FAP-to-aCAF transition and reveals a gradual increase in aCAF metagene expression towards tumor borders, forming a peritumoral “shell” pattern, (b) associates tumor adjacency with aCAF expression using tumor-distance modeling, (c) uncovers a layered spatial organization in which immune signals peak outside the aCAF-enriched zone and (d) using cross-cancer co-localization analysis, identifies macrophage and antigen-presenting cell programs, ECM remodeling and EMT signals as key features associated with aCAF-rich regions, while T-cell and B-cell activation markers are spatially depleted. These results demonstrate the power of SpatialAttractor, a co-localization analysis tool that we developed for this task (Methods) to explore spatial gene programs in the tumor microenvironment.

This multilayer pattern, consistently observed across samples in multiple cancer types, highlights a spatial relationship between aCAF distribution and immune responses, providing insights into how aCAFs contribute to tumor progression and immune exclusion leading to poor prognosis and chemoresistance.

## 2 Result

### 2.1 Spatial reconstruction of fibroblastic differentiation and proximity modeling links tumor adjacency to elevated COL11A1+ aCAF enrichment

Building on the previous single-cell RNA-seq finding that identified a transition from ASC/FAPs to aCAFs (Zhu et al.), we extended our analysis to high-definition (HD) Visium and standard Visium spatial transcriptomics to map the corresponding trajectory *in situ* across multiple cancer types. This allowed us to characterize the transition within its spatial context and to examine how it interacts with tumor cells and the surrounding tumor microenvironment (Methods). The list of standard Visium samples is presented in **Supplementary Table 1**.

**Figure 1:**
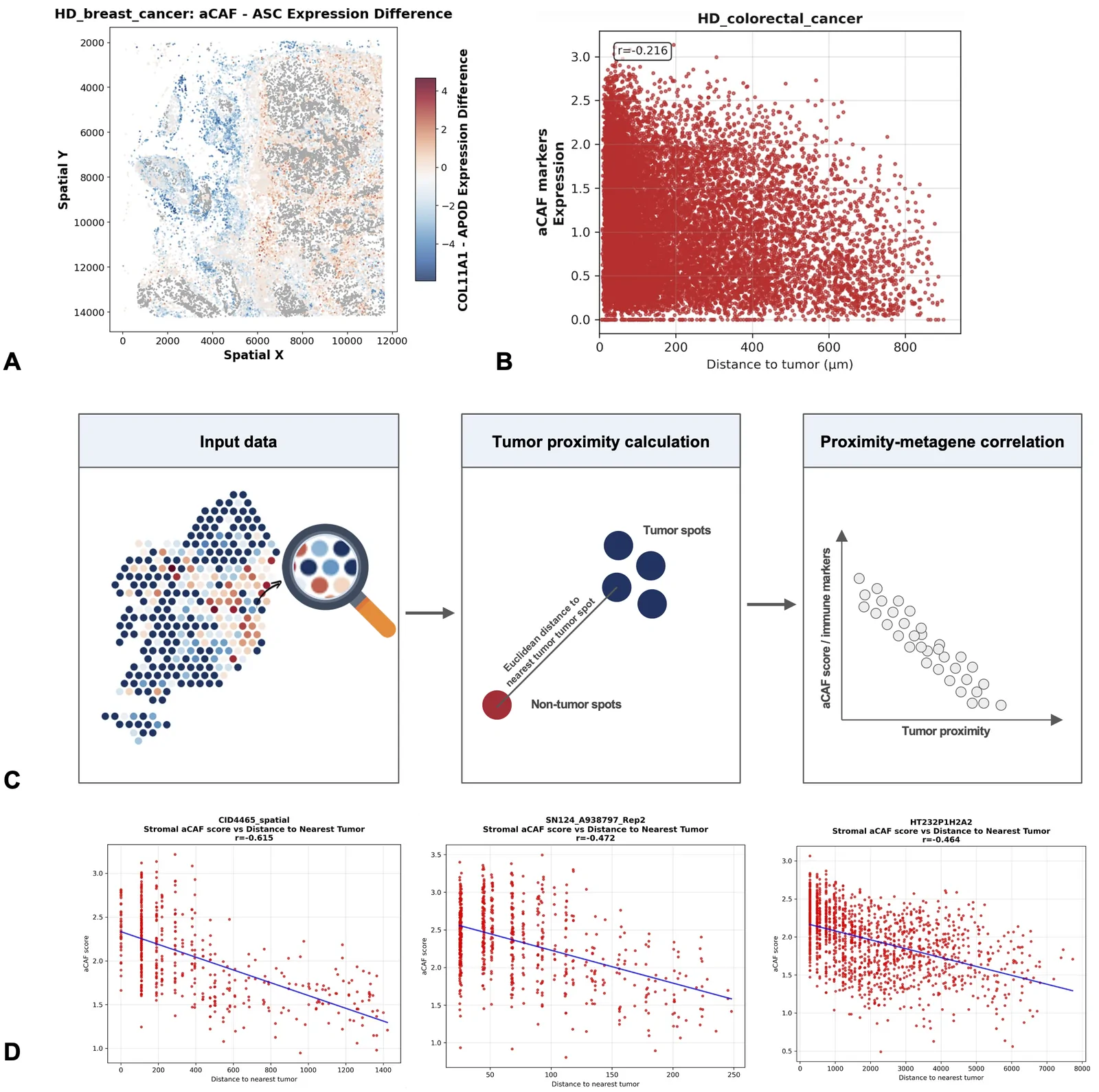
Spatial reconstruction of the ASC-to-aCAF transition and tumor-proximity modeling. **(A)** Spatial trajectory-associated change in marker expression shown as the difference between the aCAF marker COL11A1 and ASC marker APOD along the inferred ASC-aCAF transition in the Visium HD breast cancer sample. **(B)** Relationship between aCAF expression level and tumor proximity in the Visium HD breast cancer sample. Each point represents a fibroblast with its aCAF metagene score plotted against Euclidean distance to the nearest tumor cell. **(C)** The quantification workflow of the identification of tumor regions/spots, computation of stromal-spot distance to the nearest tumor spot. **(D)** Scatter plots show aCAF score versus distance to the nearest tumor spot across breast cancer, colorectal cancer, and pancreatic ductal adenocarcinoma (PDAC).

We reconstructed detailed spatial trajectories of a high-definition breast cancer spatial transcriptomics dataset (visium-hd-cytassist-gene-expression-libraries-human-breast-cancer-ff-ultima-4) using spaTrack, a spatial transcriptomics trajectory inference tool that capture differentiation topology and cell dynamics (Methods) (30). The inferred pseudotime revealed a continuous, spatially coherent progression from ASC/FAPs to aCAFs, with the late aCAF states predominantly localized in peritumoral regions (**Supplementary Figure 1**). Consistent with this trajectory, expression labels of the aCAF signature increased gradually along the inferred pseudotime, confirming the continuity of the transition (**Figure 1A**). A colorectal cancer Visium HD sample (visium-hd-cytassist-gene-expression-libraries-of-human-crc-v4) and a lung cancer Visium HD sample (visium-hd-cytassist-gene-expression-human-lung-cancer-fixed-frozen) indicate the same trend (**Supplementary Figure 1B**). We then calculated the ASC and aCAF expression difference in multiple standard Visium datasets from additional cancer types and observed a continuous ASC/FAP-to-aCAF transition across samples (**Supplementary Figure 1C**).

Given the enriched localization of late-stage aCAFs near tumor borders, we next quantitatively assessed how aCAF expression levels vary with spatial distance to tumor regions (**Figure 1B**). For each fibroblast in the breast cancer HD dataset, we computed its Euclidean distance to the nearest tumor cell and quantified the aCAF metagene signature expression (**Supplementary Table 2**) (6) (Methods) (**Figure 1B**). Fibroblasts in close proximity to tumor cells show higher aCAF scores, suggesting a spatial association between tumor proximity and aCAF enrichment. The methodology workflow of standard Visium data is demonstrated in **Figure 1C**. A negative correlation was observed between the aCAF score and tumor distance across individual stromal spots (**Figure 1D**). This spatial dependency was consistently reproduced across multiple standard Visium samples from diverse cancer types. These results demonstrate that the ASC/FAP-to-aCAF transition is not only a transcriptional trajectory but also has a distinct spatial pattern within the tissue, culminating in a shell-like aCAF structure that surrounds the tumor border.

### 2.2 Identification of spatial patterns of immune infiltration around tumor regions

Building on our previous study (6), which linked aCAF abundance to tumor stage and metastasis, we next investigated how immune programs vary with spatial aCAF proximity to tumor regions.

**Figure 2:**
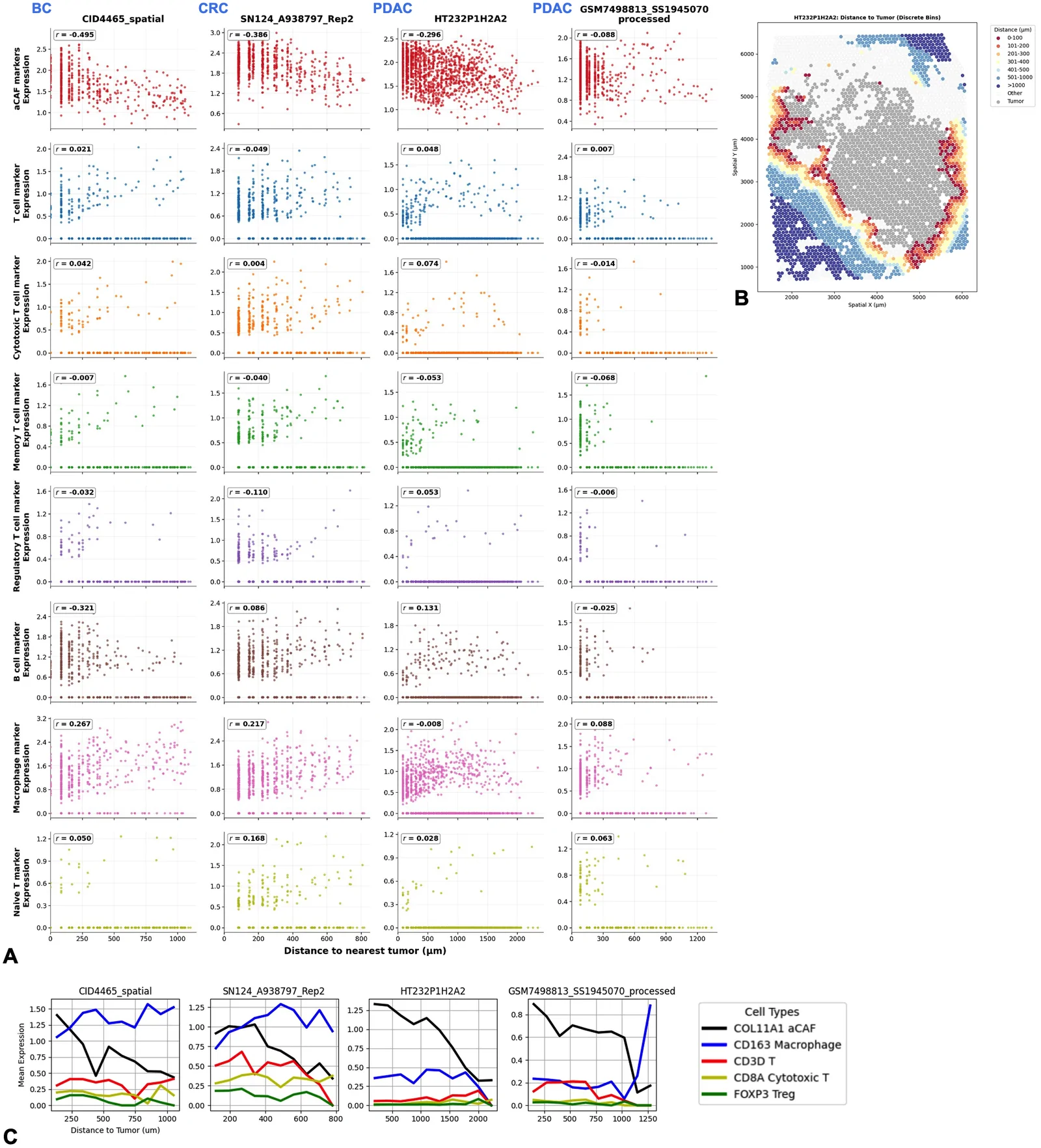
Distance-to-tumor modeling reveals different spatial trends of aCAFs and immune markers in fibroblastic regions. **(A)** Scatter plots modeling marker expression and the distance to the tumor region. Columns correspond to individual samples across cancer types. Rows show, from top to bottom, the aCAF metagene score and the immune-marker scores (**Supplementary Table 2**). Each point represents a fibroblastic spot. **(B)** Binned visualization of tumor-proximity. Fibroblastic spots are grouped into discrete distance bins from the tumor boundary, in which colors indicate the bin identity. **(C)** Plots of the mean levels of the marker expression and the distance to the tumor region.

We modeled the expression of immune and aCAF markers as a function of the distance to the nearest tumor region within stromal LUM-high clusters (**Figure 2A**). Each point in the scatterplot represents a spot identified within these clusters. Overall, the aCAF marker shows high expression levels near the tumor boundary, gradually inclined with increasing distance to the tumor cells (**Figures 2B, C**). By contrast, the T cell marker CD3D shows low expression near the tumor but tends to increase with the distance to the tumor cells, consistent with T cell infiltration into stromal regions adjacent to the tumor. The macrophage marker CD163 increases with the distance to the tumor cells as well. Specifically, in the pancreatic sample HT232P1H2A2, CD3D expression increases initially before decreasing beyond the median distance. The regulatory T cell (Treg) marker FOXP3 accumulated near the tumor boundary, suggesting that Tregs preferentially localize at the tumor boundary and co-localize with aCAFs, where they may contribute to immunosuppression. All of these samples are in moderate or high stages.

In samples without aCAF enrichment, we did not see the same spatial patterns of immune infiltration around tumor regions (**Supplementary Figure 2A**). For example, macrophage maker expression varied from sample to sample: in some datasets it stayed high across the tissue (e.g., Visium_FFPE_Human_Ovarian_Cancer), while in others it was highest close to the tumor (Visium_Human_Breast_Cancer).

From the scatter plots, we observed a marked reduction in the aCAF signature at approximately 400 µm away from the tumor boundary. To quantitatively assess this transition point, we calculated the p-values across a continuum of distance thresholds, comparing COL11A1 expression between groups defined at each threshold (**Supplementary Figure 2B**). By plotting -log(p-value) versus the tested thresholds (evaluated at 20-µm increments from 0 to 1000 µm), we found a consistent pattern across samples. All samples showed the highest -log(p-value) centered around 400 µm. Based on this, we selected 400 µm as the threshold for dividing spatial spots in subsequent analyses.

### 2.3 aCAF-high tumors show reduced infiltration of cytotoxic T cells and B cells near tumor cells

Analysis of bulk RNA-seq profiles across multiple tumor types showed a consistent negative association between aCAF abundance and CD8+ T-cell infiltration across multiple cancer types (**Supplementary Figure 3A**). Expression of the aCAF marker COL11A1 was in most cases negatively correlated with T-cell infiltration scores in a statistically significant manner.

To investigate immune exclusion in spatial datasets, we stratified samples into aCAF-high (≥20% COL11A1-positive spots) and aCAF-low (<5% COL11A1-positive spots). Samples were required to contain detectable immune populations based on markers for T cells, macrophages and B cells (≥5% CD3D-, AIF1-, or MS4A1-positive spots) and identifiable tumor regions (Methods). After filtering, 28 samples were classified as aCAF-high and 14 as aCAF-low. In the high aCAF group, the analysis includes 16 BC, 1 BCC, 1 BRCA, 2 CRC, 1 NSCLC, 1 OV, and 6 PDAC samples. In the low aCAF group, the dataset includes 8 BC, 4 CRC, and 2 NSCLC samples.

**Figure 3:**
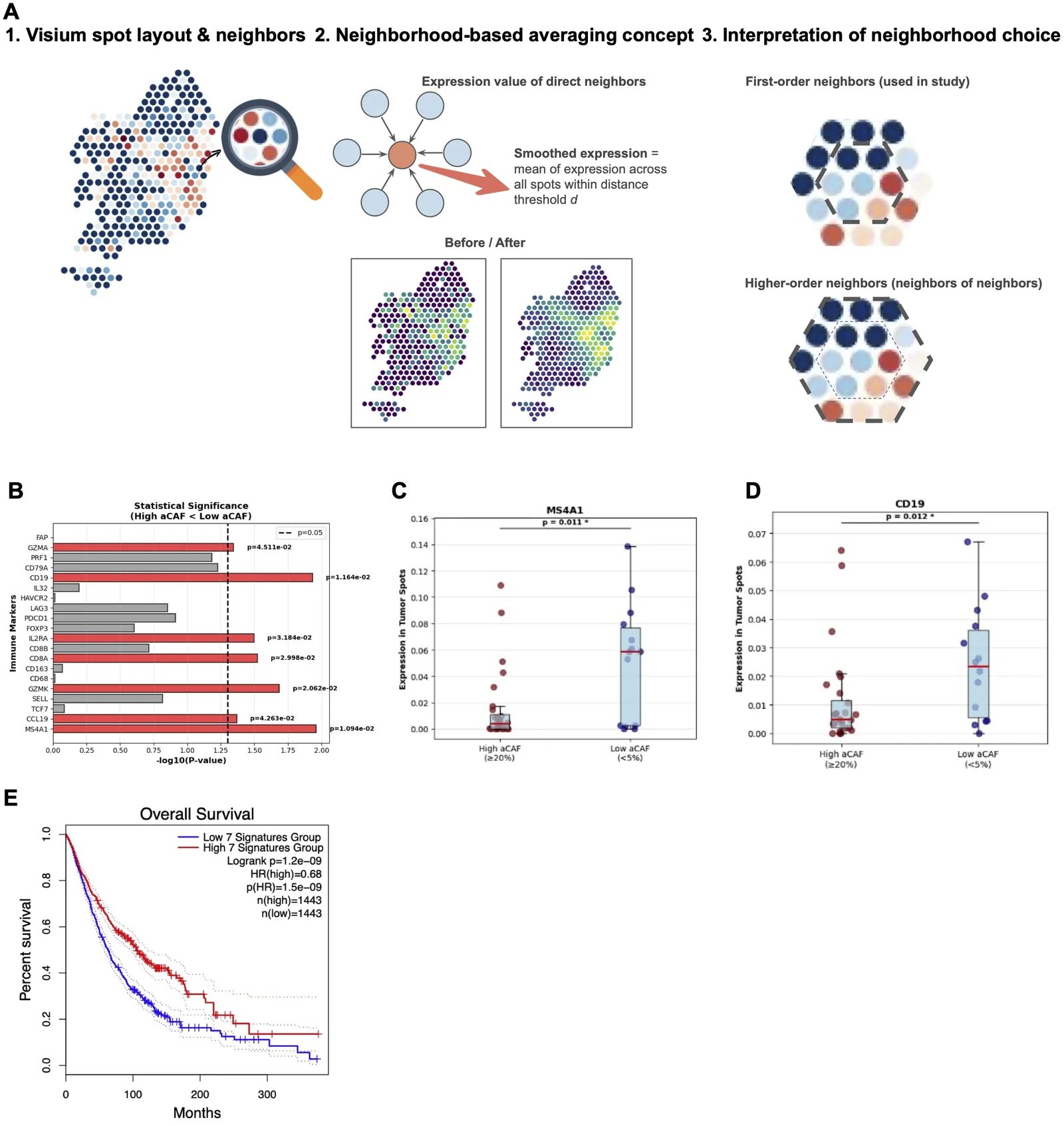
aCAF enrichment is associated with immune exclusion in tumor regions and worse clinical outcomes. **(A)** The spatial smoothing workflow for 10x Visium spatial transcriptomics data used to generate spatially smoothed gene-expression matrices for downstream analysis. **(B)** The statistical significance of various immune markers. **(C)** Boxplot of expression of MS4A1 in EPCAM-positive tumor spots from aCAF-high versus aCAF-low samples. **(D)** Boxplot of expression of CD19 in EPCAM-positive tumor spots from aCAF-high versus aCAF-low samples. **(E)** Overall survival association for a seven-gene immune marker set (**Supplementary Table 2**) across TCGA cohorts (BRCA, COAD, OV, PAAD, LUSC, SKCM; total n = 2,886). Higher expression of the immune marker set is associated with improved overall survival.

Using the spatially smoothed expression matrix (**Figure 3A**), we quantified immune gene expression within tumor-associated spots, identified by the general epithelial marker, EPCAM (**Supplementary Table 2**). Cytotoxic T-cell markers (**Supplementary Table 2**) were lower in aCAF-high tumors, indicating decreased cytotoxic T-cell activation (**Figure 3B**). Similarly, B-cell infiltration and activity were reduced, as shown by decreased expression of B cell markers (**Figures 3C, D**; **Supplementary Table 2**). CCL19, a chemokine that facilitates lymphocyte recruitment, was also significantly decreased in aCAF-high samples. A marker associated with T-cell activation and regulatory T-cell function, IL2RA, was decreased as well (**Supplementary Figure 3B**).

We analyzed these immune activation markers (**Supplementary Table 2**) across multiple TCGA tumor cohorts, including breast carcinoma (BRCA), colon adenocarcinoma (COAD), ovarian cancer (OV), pancreatic adenocarcinoma (PAAD), lung squamous cell carcinoma (LUSC) and skin cutaneous melanoma (SKCM), comprising a total of 2,886 patient samples. Our results indicate that increased expression of these immune markers is significantly associated with improved overall survival (HR = 0.68, p = 1.5 x 10^-9^). At the level of individual genes, high expression of GZMA, CD8A, GZMK, CCL19, and MS4A1 is also correlated with favorable clinical outcomes (**Figure 3E**).

These findings indicate that aCAF enrichment is associated with reduced immune infiltration and diminished activation of multiple immune populations in tumor regions. This further suggests that aCAFs may contribute to creating an immune-excluded microenvironment, which in turn leads to poor clinical outcomes.

**Figure 4:**
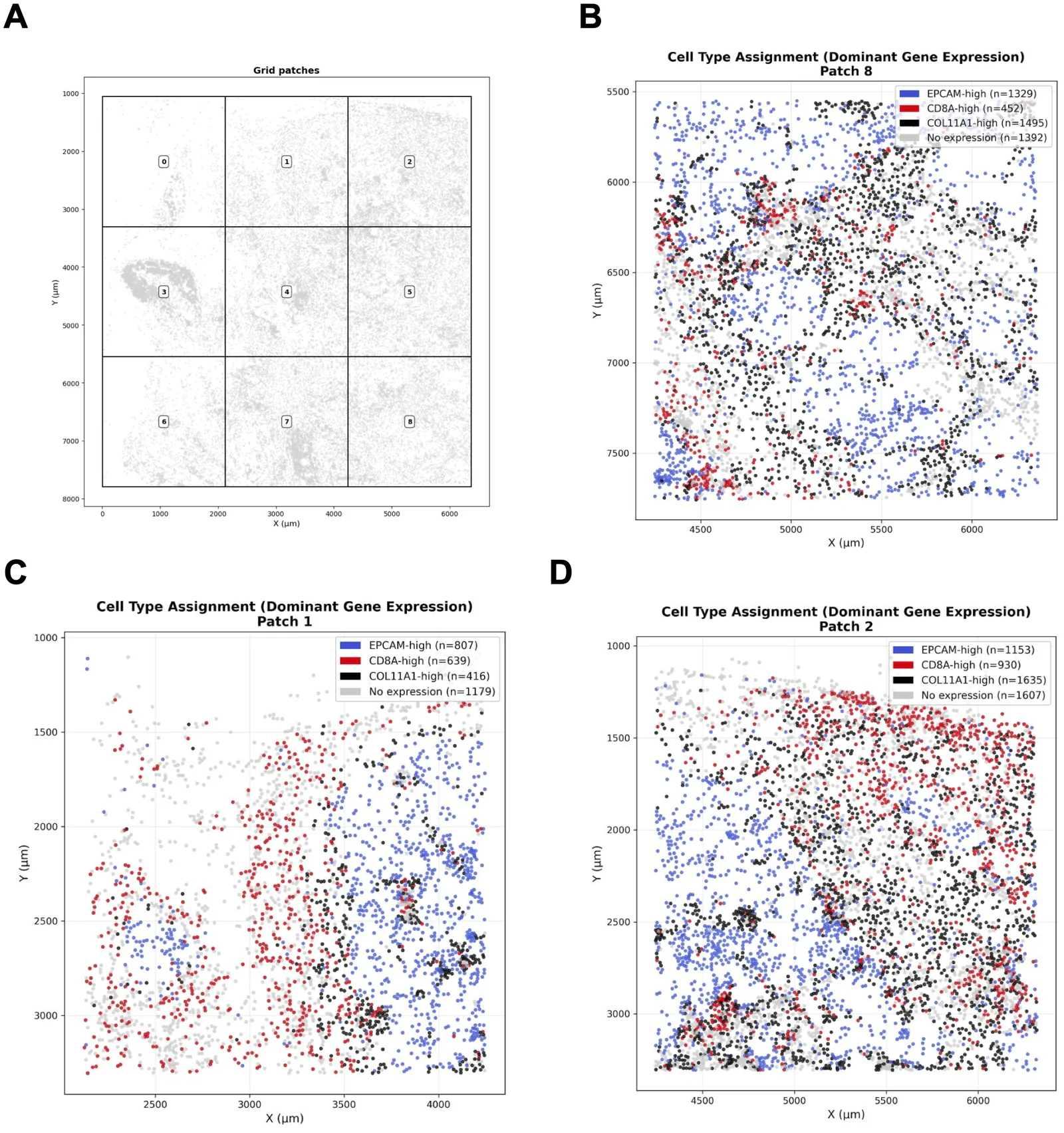
Representative single-sample spatial patch analysis illustrates patterns consistent with aCAF-associated immune exclusion. **(A)** A HD breast cancer spatial transcriptomics section divided into a 3 by 3 grid to explore local spatial relationships among tumor cells, aCAFs, and immune cells. Patches are numbered as 0-8. **(B)** Patch 8, Cell categories are color-coded as blue (EPCAM-high), red (CD8A-high), black (COL11A1-high), and grey (not classified as EPCAM-/CD8A-/COL11A1-high). **(C)** Patch 1, color-coded as **(B)**. **(D)** Patch 2, color-coded as **(B)**.

We further analyzed one representative HD breast cancer sample by dividing it into a 3x3 grid to visualize local spatial relationships among tumor cells, aCAFs, and immune cells (**Figure 4A**). Patches, such as patch 8, showed that immune cells were co-localized with aCAFs, and immune cells could not infiltrate the adjacent tumor region, suggesting that aCAFs may act as a barrier (**Figure 4B**). Patch 1 shows a clear layered structure, with aCAFs surrounding the tumor region and immune cells occupying a second outer layer (**Figure 4C**). In patch 2, aCAFs are located outside the tumor but still co-localize with immune cells; immune-cell abundance increases with greater distance from the tumor boundary (**Figure 4D**).

We identified 77 samples that have low COL11A1 expression (<10% COL11A1-positive spots out of 142 total samples; see Methods). For each sample, we assessed genes co-localized with the epithelial-tumor marker EPCAM. We selected the three samples with the highest T cell marker (CD3D) and assigned each spot to one of three categories (COL11A1-high, EPCAM-high, or immune-high) according to the dominant expression levels of the markers (**Supplementary Table 2**). The figures show that these samples lack aCAF, having noticeable immune infiltration (**Supplementary Figures 4A–C**).

### 2.4 Cross-cancer co-localization analysis highlights ECM stiffness, lipid-associated macrophage, and EMT signals

To identify cell populations and cellular programs that preferentially co-localize with aCAFs, we analyzed 64 spatial transcriptomics samples spanning breast, ovarian, pancreatic, colorectal, and lung cancers having high COL11A1 expression (>10% COL11A1-positive spots out of 142 total samples using the association measures of Mutual Information (MI), Spearman’s correlation coefficient and Moran’s I; Methods). For each sample, we applied spatial smoothing and computed MI between COL11A1 and all other genes and ranked genes based on their associations with COL11A1 expression and selected the top 2000 genes for further analysis.

A cross-cancer pattern emerged in the ranking list: following aCAF and other fibroblast markers (**Supplementary Table 3**), antigen-presenting cell and macrophage-related genes (HLA-DRA, CD68, CD163) ranked high on the ranked lists, indicating frequent co-localization of aCAF with macrophage-rich regions. Many of the consistently co-localized genes mapped to a TREM2 (observed in 36, 37, 35 among 64 cases for MI, Spearman’s correlation, Moran’s I), GPNMB (31–33) lipid-associated macrophage (LAM) module (34, 35), indicating that the lipid-associated subpopulation of macrophages were enriched in the region of interest followed by T cell markers, B cell markers, and cytotoxic T cell genes (**Supplementary Table 2**), suggesting that immune cell proximity decreases as spatial distance from aCAF-rich spots increases.

To further validate these stromal-LAM interactions, we ranked genes based on TREM2. Genes with high MI to TREM2 exhibited a concordant ordering of macrophage, aCAF, fibroblast, T cell, cytotoxic T cell, and B cell markers. This result confirms the spatial co-localization pattern observed in COL11A1. Ranking genes by their association with TREM2 across multiple cancer types reveals a spatial co-localization pattern enriched for MHC class II antigen-presentation genes (HLA-DOA, HLA-DRA, HLA-DPA1, HLA-DPB1, HLA-DMB, HLA-DQB1, CD74; **Supplementary Table 4**), along with complement components (C1QA, C1QB, C1QC) and lipid-related genes (APOE, APOC1). This gene list overlaps with the lipid-associated macrophage signature (3CA, https://www.weizmann.ac.il/sites/3CA) previously reported by the Weizmann Institute, indicating there is a “lipid-associated macrophage” state in various tumor types. We investigated the enriched programs of the top 30 shared genes (**Supplementary Table 4**) against the 3CA signatures using MSigDB (36) and identified the strongest enrichment for “lipid-associated macrophage” program (FDR q value = 3.24 ×10^-18^).

Genes COL11A1, COL10A1, COL5A1, COL5A2, INHBA, THBS2, COL12A1, FN1, ITGA11 and LUM were the ones consistently included with detection count 64 across all samples using any of the three association metrics (**Supplementary Table 3**). Most of these genes are among the top ten aCAF markers shown in **Supplementary Table 2**. Key regulators of ECM stiffness are also represented in the dataset, including members of the LOX family, such as LOXL1 (60, 62, 60), LOX (37–39), and LOXL2 (37, 38, 40). They play important roles in the process of collagen rearrangement (41). The co-localization analysis further indicates that EMT-related signals are enriched and localized near aCAFs. Several EMT-associated markers are found within the surrounding neighborhood, including ZEB2 (42–44), TWIST1 (32, 43, 45), ROR2 (42, 45, 46), RUNX2 (31, 47, 48), ITGA5 (49–51), ITGAV (31, 50, 50), and DAB2 (31, 31, 52) (42–46, 53, 54). These findings reveal a spatial organization across cancer types in which aCAFs co-localize with LAMs, ECM-remodeling factors, and EMT programs, forming a coordinated interaction between stromal, immune, and epithelial compartments to shape the tumor microenvironment.

## 3 Discussion

In this study, we provide a comprehensive spatial map of the ASC/FAP-to-aCAF transition across multiple cancer types, revealing that transition to aCAF is a spatially programmed event driven by tumor proximity. By integrating HD and standard 10X Visium spatial transcriptomics, we identified a consistent “shell-like” architecture where COL11A1+ aggressive CAFs surround the tumor along with immuno-suppressive cells, implying a “barrier” that restricts infiltration of the cytotoxic effector immune cells. These findings provide a quantitative spatial framework for understanding immune exclusion and the clinical failure of immunotherapies in stroma-rich “cold” tumors (32), which may shed light on cellular interactions in the TME as well as lead to novel biomarkers and drug design for cancer immunotherapy. Beyond the biological insights, we introduce SpatialAttractor, a toolkit for measuring spatial gene co-expression in multiple metrics within their tissue context. We included all three metrics in the toolkit.

Our work has several limitations, which should be considered. First, our analysis is based on 2D tissue sections although the tumor “shell” is a 3D structure. A 2D slice may sometimes misrepresent the true Euclidean distance to the nearest tumor. Second, spatial transcriptomics measures RNA abundance and cannot directly establish causality. Tumor proximity may be both a driver and a consequence of stromal remodeling. Third, although a higher aCAF abundance showed a directionally consistent association with lower immune marker expression across cancer types, the magnitude of this association varied. Future studies using larger and more cancer-type-balanced cohorts will be needed to determine the biological basis of this heterogeneity and whether tumor-specific microenvironmental features modulate aCAF-associated immune exclusion. Nevertheless, complementary experimental evidence supports a functional role for the ASC/FAP-to-aCAF transition in tumor progression. Our recent *in vitro* and *in vivo* studies using the pancreatic cancer cell line Capan-1 demonstrate that inhibition of ASC-derived aCAFs suppresses Capan-1 cell migration and invasion. As an example, tumors in SFRP4-knockout mice, in which the ASC/FAP-to-aCAF transition is inhibited, show reduced desmoplasia, less epithelial dedifferentiation, decreased growth rate, and inhibited progression to metastasis (47).

## 4 Methods

### 4.1 Dataset preparation and filtering

We obtained a total of 142 samples from publicly available datasets and quantified the proportion of spots expressing genes of interest (**Supplementary Table 1**). All subsequent analyses were conducted on the subset of samples containing defined regions of interest (ROIs). For each sample, we computed the proportion of spots having expression levels above 1 for each gene of interest (e.g., APOD, COL11A1, and EPCAM).

For downstream analyses, we applied a series of sample-level filters to ensure the robust detection of fibroblast population, tumor regions, and immune infiltration. We used spot-level abundance thresholds corresponding to 5%, 10%, or 20% of spots expressing a given marker, representing low, intermediate, and high inclusion criteria, respectively.

Samples included in the spatial ASC/FAP-to-aCAF transition, distance-to-tumor analysis (**Figure 1D**; **Supplementary Figure 1**; **Figure 2**; **Supplementary Figure 2**) were required to have ASC, aCAF, and tumor populations, defined as having ≥10% of spots expressing APOD (APOD_pct_gt1 ≥ 10), ≥10% expressing COL11A1 (COL11A1_pct_gt1 ≥ 10), and ≥20 expressing EPCAM (EPCAM_pct_gt1 ≥ 20), with a minimum tissue size of 1,200 spots. We used intermediate APOD and COL11A1 thresholds to have enough cells in both stromal states for modeling their spatial transition, while a stricter EPCAM threshold ensured clearly defined tumor compartments for reliable distance-based analysis. For the MI list assessment (**Supplementary Tables 3**, **4**), we kept samples with at least 10% COL11A1-positive spots, resulting in 64 spatial transcriptomics samples. This threshold was chosen to maximize the number of informative samples while maintaining robust detection of COL11A1-positive spots for co-localization analysis. For the sample-level comparative analysis (**Figure 3**), we identified two groups based on combined fibroblasts, tumor, and immune content. Both groups required ≥20% EPCAM-positive spots and evidence of immune infiltration, defined as ≥5% of spots positive for at least one representative immune marker (CD3D, AIF1, or MS4A1). The high-aCAF group had ≥20% COL11A1-positive spots, while the low-aCAF group had <5%.

### 4.2 Trajectory inference

Trajectory inference on spatial transcriptomics tumor tissue data was performed in Python using Scanpy (v1.9.8) and spaTrack (v0.1.1). Raw gene expression matrices and spatial metadata were loaded and preprocessed, including normalization, log transformation, filtering of lowly expressed genes and clustered based on graph-based methods. Spatial trajectories were then inferred with spaTrack, and cellular dynamics were characterized using pseudotime estimation and vector field analysis. The analysis followed the spaTrack tutorial for intrahepatic cholangiocarcinoma cancer (https://spatrack.readthedocs.io/en/latest/notebooks/02.ST_data_of_Intrahepatic_cholangiocarcinoma_cancer.html#).

### 4.3 Preprocessing for standard and high-definition spatial transcriptomics

Spatial transcriptomic data generated using 10x Genomics Visium and Visium HD platforms were processed using a unified Python/Scanpy workflow. For each dataset, mitochondrial genes (prefix “MT-”) were annotated and per-spot quality control (QC) metrics were computed with *scanpy.pp.calculate_qc_metrics*. A slide-based adaptive QC strategy was applied, retaining only on-tissue positions (in_tissue = 1), removing spots with low transcript complexity (< 200 detected genes), and limiting the total UMI counts to a slide-based adaptive window defined as the greater of 1,000 UMIs or the 1st percentile (lower bound) and the greater of 35,000 UMIs or the 99th percentile (upper bound). Spots with high mitochondrial content (≥ 20% mitochondrial reads) were excluded. This adaptive filtering approach preserves biologically meaningful spatial heterogeneity while minimizing technical noise. After filtering, counts were normalized to a constant library size and log-transformed (*scanpy.pp.normalize_total* and *scanpy.pp.log1p*). Dimensionality reduction and clustering were performed using PCA, k-nearest-neighbor graph construction, UMAP embedding and Leiden community detection (*scanpy.pp.pca, sc.pp.neighbors, scanpy.tl.umap, scanpy.tl.leiden*).

Visium HD data were processed using a custom reader that imports expression matrices and spatial metadata generated by the 10x Genomics pipeline. Raw gene expression counts were loaded from the raw_feature_cell_matrix.h5file. High- and low-resolution tissue images, along with platform-provided scale factors, were retrieved from the spatial directory and stored in the AnnData structure to enable downstream spatial visualization and scaling. Cell-level segmentation information was imported from the Space Ranger-generated GeoJSON file, and polygon centroids were used to define the spatial coordinates of each segmented cell. Mean cell area was used to estimate a segmentation-based spot diameter in pixels, which was stored in the spatial metadata. Expression counts were then normalized to a constant library size and log-transformed. Graph-based clustering results produced by Space Ranger HD (gene_expression_graphclust) were imported, with cells lacking valid cluster assignments removed.

Physical spatial coordinates were computed by converting pixel-based positions to micrometers using platform-specific scale factors. For standard Visium, the physical scale was estimated from the known 55 µm spot diameter, using the relationship microns_per_pixel = 55 μm/spot_diameter_fullres. Afterward, all spatial coordinates were multiplied by this factor to obtain micrometer-resolved embeddings. For Visium HD, which provides a direct pixel-to-micron conversion factor in the output metadata, physical coordinates were obtained by multiplying each cell’s centroid position by the platform-supplied microns_per_pixel value. This ensured that both datasets were placed on a consistent physical scale suitable for downstream spatial distance analyses.

### 4.4 Identification of fibroblasts and cancer cell populations

For each sample, epithelial and fibroblastic clusters were automatically identified using a marker-based cluster expression profiling approach. For each cluster, we computed the mean expression of a specified marker gene and standardized these values across clusters by calculating the global mean and standard deviation. Clusters were classified as high-expressing when their mean expression exceeded the overall cluster mean by at least 0.5 standard deviations. Applying this criterion, EPCAM-high clusters were annotated as tumor regions, and LUM-high clusters were annotated as fibroblast regions. EPCAM and LUM were selected because they are broadly applicable markers for our multi-cancer analyses.

In the case of the CRC-HD sample, we incorporated prior knowledge of tumor boundaries and manually assigned tumor regions based on the reference annotation shown in **Figure 4D** of Oliveira et al. (31).

### 4.5 Quantifying distance to the nearest tumor and evaluating gene expression

To quantify the spatial relations between fibroblasts and tumor regions, we calculated the Euclidean distance from every spot or cell to the nearest tumor-annotated location using micrometer-scale spatial coordinates. Tumor spots or cells were identified following the cluster-label expression-profiling strategy (Methods, Identification of fibroblasts and cancer cell populations). For each spot, we calculated the minimal distance to any tumor coordinate:

$d_{i}=\underset{j}{\min}∥x_{i}-x_{j}^{\left(\text{tumor}\right)}{∥}_{2}$This generated a continuous measure of tumor proximity across the tissue. Gene expression was evaluated using markers representing aCAF and immune states. aCAF scores were assessed using the top ten genes listed in Kim et al. (6): COL11A1, THBS2, COL10A1, COL5A2, INHBA, LRRC15, COL5A1, VCAN, FAP, and COL1A1. Immune markers we used are listed in **Supplementary Table 2**.

### 4.6 Spatial transcriptomic smoothing of gene expression and co-localization

We proposed a “spatial transcriptomic smoothing” approach that incorporates local spatial context with gene expression to capture co-localization patterns. Instead of relying entirely on normalized expression values from individual Visium spots, we replaced each spot’s expression profile with the average expression of the spot and its direct neighbors. On the Visium platform, a non-edge spot typically has six adjacent neighbors. Thus, the averaged value represents the expression across seven spots in total. This neighborhood-based averaging is designed to more accurately capture co-localization patterns by integrating information from the surrounding tissue microenvironment. While the method can be generalized to second-order or higher-order neighborhoods (i.e., neighbors of neighbors), we observed that including direct neighbors provided the most interpretable results. Expanding to higher-order neighborhoods introduced excessive smoothing, thereby reducing local spatial variation.

For each spot (i, j), the smoothed expression value ${\tilde{g}}_{i,j}$ was defined as the mean expression across the spot itself and all neighboring spots with a specified distance threshold *θ*:

${\tilde{g}}_{i,j}=\frac{1}{\left|N_{i,j}\right|}\sum_{(k,l)\in N_{i,j}}g_{k,l}$ $N_{i,j}=\{(k,l):d((i,j),(k,l))\leq \theta \}$where *θ* is the distance threshold, *g_k,l_* is the gene expression at spot (*k*, *l*), and |*N_i,j_*| is the number of neighbors in the set $N_{i,j}$https://www.codecogs.com/eqnedit.php?latex=N_%7Bi%2Cj%7D – 0. In this study, *θ* was defined as the spacing between two immediately adjacent spots on the Visium array.

### 4.7 Mutual information estimates on the neighborhood-smoothed matrix

Mutual information (MI) has been adopted as a general measure of the correlation between variables for its capability to capture non-linear correlations in gene expression data (33, 48). We adopted the definitions of MI estimator based on B-spline functions with the parameters (bins = 6, order = 3) adopted from a systematic evaluation on gene expression data proposed by Daub et al. (48) to quantify gene-gene associations within the neighborhood-smoothed expression matrix, we implemented the FastMICalculateNumba() module to estimate mutual information between the smoothed expression profile a seed gene (e.g., COL11A1) and all other genes in the matrix using the MI estimator (48, 50). We divided the estimated MI values of two genes by the maximum of the estimated MI values of each gene to enable comparison across genes. As MI is a non-negative quantity, we defined the sign of the estimated MI value as the sign of Pearson’s correlation coefficient between two genes (50).

### 4.8 Co-localization analysis using spatialAttractor toolkit

For each sample, co-localization analysis was performed using the spatialAttractor framework. Raw spatial transcriptomics data were first processed using the spatialTool() function, which performed standard preprocessing and quality control to generate an expression matrix. To incorporate spatial context and reduce local noise, gene expression values were smoothed using the FuzzyAverage() module, which computes a neighborhood-weighted fuzzy average across spatially adjacent spots (n_neighs=6, coord_type= “grid”, include_self=True). The resulting smoothed matrix was then used for downstream association analysis.

Gene-gene associations were quantified by estimating the mutual information and Spearman’s correlation coefficient (52) between the smoothed expression profile of a seed gene (e.g., COL11A1) and all other genes in the matrix using the FastMICalculateNumba() module. For each sample, genes were ranked according to their MI scores in terms of the seed gene, resulting in a ranked list of genes whose spatially smoothed expression patterns were most strongly associated with COL11A1. These ranked gene lists were subsequently aggregated across samples for downstream analysis.

Spearman correlation was calculated between the smoothed expression profile of the seed gene and each other gene to capture monotonic association, complementing MI’s sensitivity to non-monotonic dependence. Moran’s I (51) was calculated for the seed gene and for each top-ranked gene individually to confirm that genes identified as co-patterned with the seed gene also exhibit significant spatial autocorrelation in their own expression, rather than treating Moran’s I as an alternative seed-gene ranking method. Reporting these statistics together allows genes with strong, consistent support across measures to be distinguished from those supported by MI alone.

### 4.9 Co-localization permutation test

To determine whether genes truly co-localize with COL11A1, rather than appearing together by chance or because they are simply highly expressed, we used a permutation-based statistical approach (**Supplementary Figure 2B**). For selected samples for co-localization analysis, we created a null model by randomly shuffling the spatial coordinates 20 times while keeping all gene expression values unchanged. This breaks any real spatial structure preserving each gene’s overall expression pattern. For every shuffled dataset, we recalculated the smoothed spatial matrix and MI between COL11A1 and all other genes.

Then, we compared the observed MI to the null distributions to calculate p-values, defined as the fraction of permuted MI values that were equal to or higher than the real value. To account for testing thousands of genes, we applied the Benjamini-Hochberg false discovery rate (FDR) procedure (alpha = 0.05). Genes with FDR-adjusted p < 0.05 were considered to have significant co-localization. Finally, we identified genes that remained significant across multiple samples to highlight robust, biologically meaningful spatial patterns rather than sample-specific effects.

## Data Availability

The original contributions presented in the study are included in the article/**Supplementary Material**. Further inquiries can be directed to the corresponding author.
