# Supplementary material for: Spatial dynamics of cancer-associated fibroblasts links fibroblastic differentiation to immune exclusion and tumor progression
Source: Front Immunol. 2026 Aug 28;17:1886779. doi: 10.3389/fimmu.2026.1886779 (PMC13562289; doi:10.3389/fimmu.2026.1886779)
Supplement: Supplementary file 1 [file Supplementaryfile1.docx]

Supplementary Figures

**Spatial dynamics of cancer-associated fibroblasts links fibroblastic differentiation to immune exclusion and tumor progression**

**Lingyi Cai^1, 2, †^, Tai-Hsien Ou Yang^1, 2, †^, Dimitris Anastassiou^1, 2, 3,^ ***

^1^ Department of Systems Biology, Columbia University, New York, NY, USA

^2^ Department of Electrical Engineering, Columbia University, New York, NY, USA

^3^ Herbert Irving Comprehensive Cancer Center, Columbia University, New York, NY, USA

*** Correspondence:**
Dimitris Anastassiou
[d.anastassiou@columbia.edu](mailto:d.anastassiou@columbia.edu)
† These authors contributed equally to this work

**Supplementary Figure 1.** Spatial-expression trajectories and APOD-COL11A1 patterns across cancer types. (**A)** spaTrack pseudotime trajectories across Visium HD datasets from breast, colorectal, and lung cancers. Color indicates inferred pseudotime along the reconstructed ASC-aCAF trajectory. (**B)** The difference visualization (COL11A1-APOD) in a Visium HD colorectal cancer sample and a Visium HD lung cancer sample show the same trend observed in breast cancer. **(C)** COL11A1-APOD expression difference in standard Visium samples across three cancer types (breast cancer, colorectal cancer, and PDAC).

**A**

**B**

**C**

**Supplementary Figure 2.** Samples lacking aCAF enrichment show distinct distance-dependent patterns. **(A)** Scatter plots of marker expression versus distance to the nearest tumor region for samples without enriched aCAFs (sample IDs: GSM6433589_093C, GSM6433590_093D, SN048_A416371_Rep1, SN123_A798015_Rep1, SN124_A798015_Rep2, P17, HT242P1H4-S1Fc1U1Z1B1_1, HT264P1-S1H2Fc2U1Z1Bs1-H2Bs2-Test, Visium_FFPE_Human_Ovarian_Cancer, CytAssist_FFPE_Protein_Expression_Human_Breast_Cancer, V1_Breast_Cancer_Block_A_Section_2 and Visium_Human_Breast_Cancer), showing patterns distinct from aCAF-high samples in Figure 2. **(B)** Optimal cut-off of distance identification for downstream analysis. For each sample, COL11A1 expression was compared between spots grouped by different distance cutoffs (tested every 20 µm from 0-1000 µm). Plots show -log(p-value) as a function of the tested threshold. **(C)** Plots of the mean levels of the marker expression and the distance to the tumor region.

**A**

**B**

**Supplementary Figure 3.** COL11A1 is negatively associated with immune infiltration and immune-marker expression in tumor regions. **(A)** Pan-cancer partial correlation analysis between COL11A1 expression and estimated immune infiltration using TIMER 3.0. Rows represent cancer types and columns represent CD8+ T-cell infiltration estimates computed by different algorithms. The heatmap color indicates partial correlation coefficients, showing a consistent negative association between COL11A1 expression and CD8+ T-cell infiltration across various tumor types. **(B)** Immune-marker expression in tumor-associated EPCAM-positive spots (EPCAM expression > 1) stratified by aCAF abundance. Boxplots show the expression of CCL19, GZMK, CD8A, IL2RA, and GZMA by aCAF abundance.

**A**

**B**

**C**

**Supplementary Figure 4.** Representative COL11A1-low samples show limited aCAF signal and prominent immune infiltration. **(A)** Lung cancer sample P19. Blue shows tumor-enriched (EPCAM-high) spots, black indicates aCAF-enriched (COL11A1-high) spots, and red indicates immune-high spots defined by the immune markers (Table S3). **(B)** Breast cancer sample GSM6433623_397D. Color scheme and immune categories as Figure S4A. **(C)** Breast cancer sample GSM6433609_120B. Color scheme and immune categories as Figure S4A.
